# Comparison of Cerebrospinal Fluid Collection Through the Proximal and Distal Port Below the Overflow System from an External Ventricular Drain

**DOI:** 10.1007/s12028-022-01615-y

**Published:** 2022-10-13

**Authors:** Christina Barbara Kinast, Michael Paal, Uwe Liebchen

**Affiliations:** 1 Department of Anesthesiology, University Hospital, Ludwig Maximilian University Munich, Marchioninistrasse 15, 81377 Munich, Germany; 2 Institute of Laboratory Medicine, University Hospital, Ludwig Maximilian University Munich, Munich, Germany

External ventricular drains (EVDs) have been used for acute relief of hydrocephalus and also for reliable monitoring of intracranial pressure and are thus one of the most common neurosurgical interventions. An EVD can be inserted almost regardless of the cause of the hydrocephalus, for example, in subarachnoid hemorrhage (SAH), in intraventricular hemorrhage, in brain tumors, in shunt failure, or in the case of a stroke. However, any EVD insertion is associated with the risk of infection, with typical ventriculostomy-related infection (VRI) rates of approximately 10% described [1]. To reduce the risk of VRI, it is recommended to remove the EVD as early as possible and to use antimicrobial impregnated catheters. In addition, cerebrospinal fluid (CSF) sampling should be strictly indication-related and restrictive (i.e., avoiding daily sampling), given that manipulations at the proximal EVD port may increase the risk of infection [1, 2]. However, under certain circumstances, regular diagnostic sampling is indispensable, for example, in anesthetized patients in the intensive care unit where clinical assessment is not possible, and signs of a VRI might be missed if CSF is not frequently analyzed [3].

CSF samples are typically drawn at the proximal port closest to the head (see Fig. 1). The CSF can also be collected via the distal overflow system, in which the draining fluid drips off into a collection chamber after passing through a refined filter device, minimizing the risk of ascending infections. However, this CSF supernatant is usually disposed with a drainage bag. So far, there is only a single study investigating CSF collection from the proximal port and distal port (below the overflow chamber) [4]. In the corresponding study, Wong [4] investigated differences between CSF samples obtained from proximal and distal collection sites for protein, glucose, cell count, and culture in 47 patients and employed Pearson’s correlation coefficient to compare both sampling sites. The present study aimed to evaluate whether distally obtained CSF supernatant provides comparable test results for a variety of laboratory parameters that are used in clinical routine. In addition to the standard parameters, such as protein, glucose, lactate, cell count, and CSF cytology (with stained cytospin preparations), we investigated several biomarkers that can be requested from clinical laboratories for diagnostic workup, including interleukin 6 (IL-6), ferritin, protein S100, and neuron-specific enolase (NSE). Although several clinical conditions, including bacterial infections, are associated with increased IL-6 levels in CSF [5], ferritin is a sensitive diagnostic test in SAH. In contrast, elevated protein S100 and NSE levels in CSF can be considered as evidence of the degree of brain damage in neurodestructive conditions. Given that red blood cells also contain large amounts of NSE hemolysis may cause a marked increase of NSE in CSF samples. For this reason, we also examined CSF samples with blood, as they are typically present in intracranial hemorrhage CSF specimens. They were only withdrawn from distal overflow reservoirs that had previously been completely emptied to rule out distorted cell counts due to cell settling.

**Fig. 1:**
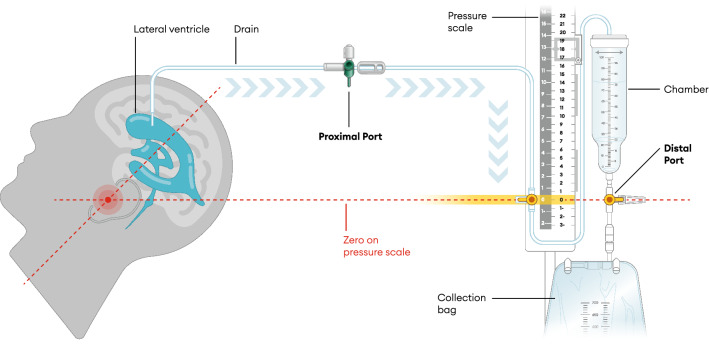
Structure of the extraventricular drainage system

This prospective, monocentric study in two intensive care units (neurological and neurosurgical) at the tertiary care hospital of the University of Munich, Ludwig Maximilian University, was approved by the responsible local Ethics Committee (registration number: 20-169, NCT04426383). Written informed consent was given by all patients or their legal representatives. Withdrawal of diagnostic samples from the proximal port of the VentrEX EVD (Neuromedex, Hamburg, Germany) was synchronized with CSF supernatant collection from a distal port (time interval ≤ 1 h). The overflow reservoir was first completely emptied, and the CSF supernatant was withdrawn after a fill state of 2 ml. All CSF samples were taken by foot to the clinical laboratory for immediate analysis. The measurement results were compared by applying graphical (classical goodness-of-fit plots) and statistical evaluation methods (Lins concordance of correlation coefficient [CCC]) with the R programming language (version 4.0.2) using the R package DescTools. A CCC of > 0.8 demonstrated that diagnostic CSF sampling from the proximal and distal reservoir ports are analytically equivalent [6].

A total of 20 patients were included in the study (11 men, 9 women), with up to two samples per patient being subjected to the comparative measurements (35 sample pairs in total). The median age of the patients was 56 years, and 15 patients suffered from SAH, 2 patients from postoperative hydrocephalus, and 1 patient each from intracerebral hemorrhage, subdural empyema, and traumatic brain injury. The agreement of the comparative measurements is illustrated in goodness-of-fit plots in Fig. 2.

**Fig. 2:**
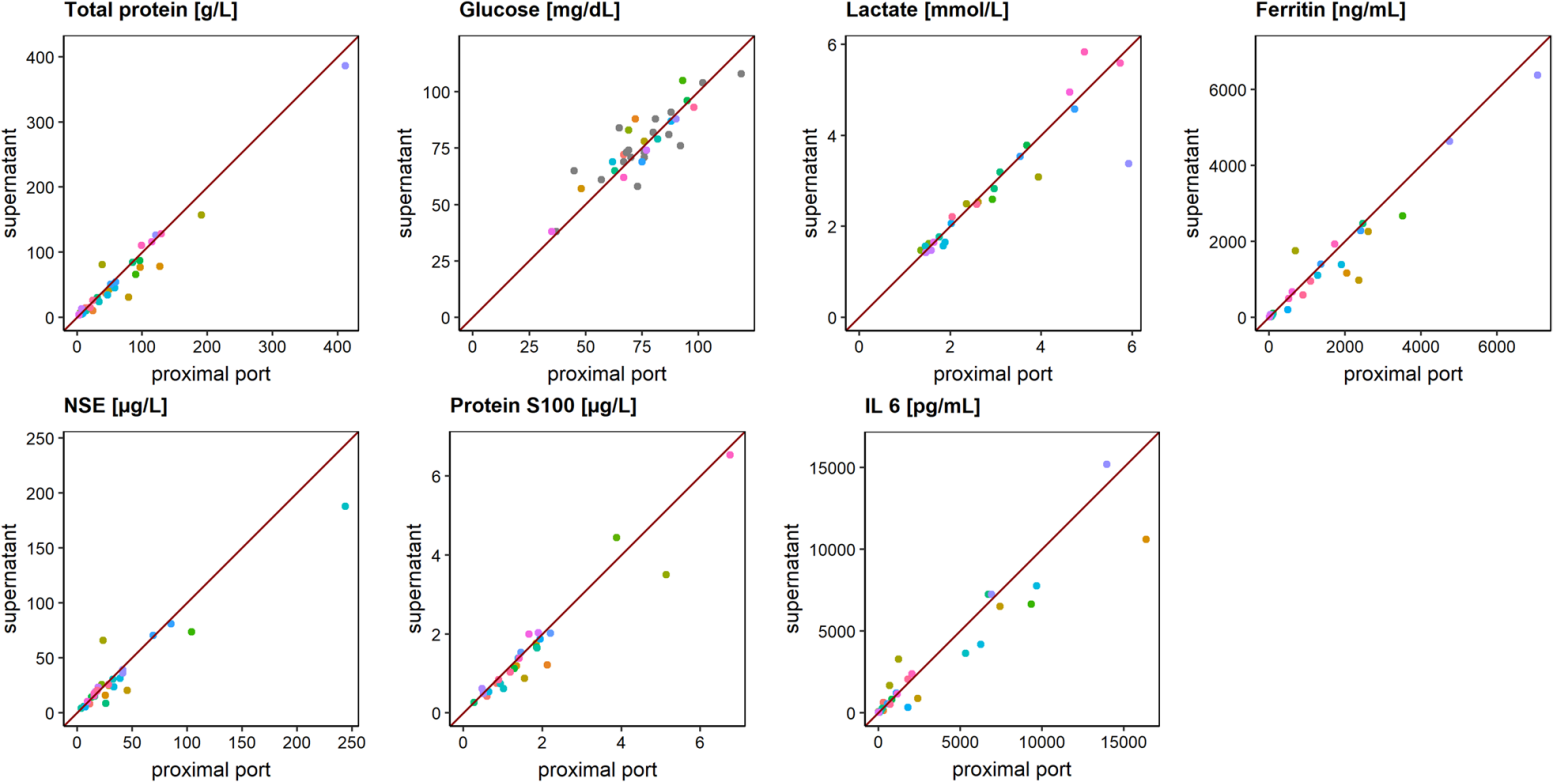
Goodness-of-fit plots comparing clinical laboratory parameters from samples drawn from the proximal port of an external ventricular drain and from the supernatant of an external ventricular drain system. Colors of the points indicate the different patients. IL-6, interleukin 6; NSE, neuron-specific enolase

None of the biochemical laboratory parameters showed a structural deviation from the line of identity over the investigated concentration range. In accordance with the graphical results, the numerical results revealed a CCC ≥ 0.88 for all biochemical analytes indicating a good agreement between the direct sampling from the proximal port and the analysis of the supernatant obtained from the distal port below the collection chamber (see Table 1). It must be noted that the population consisted mainly of patients with cerebral hemorrhage and that artifactual hemolysis can produce falsely elevated values, in particular for the analyte NSE. However, in our study, there was no evidence of compromised NSE measurements, possibly due to a prompt collection under study conditions. However, one must exercise caution when CSF supernatant is collected over a long period, as in vitro hemolysis in the EVD device cannot be ruled out. Protein S100, which—like NSE—is regularly used as a marker for cell death, will present with falsely higher results only on severe hemolysis. Given that interference is unlikely, protein S100 analysis from the supernatant can be considered reliable almost without exception. Our results are in accordance with the results by Wong [4] in 47 patients, who found a high correlation between glucose and protein concentrations from the proximal and the distal EVD port. As it might be expected—based on Wong’s [4] results—we found a good agreement for other biochemical laboratory parameters, as well.

**Table 1:** Lin’s concordance correlation coefficient for the comparison of proximal and supernatant sampling

Parameter	Mean (SD)	Lin’s concordance correlation coefficient (95% CI)
Total protein (g/l)	63.2 (76.6)	0.97 (0.95–0.99)
Glucose (mg/dl)	74.5 (17.8)	0.88 (0.77–0.93)
Lactate (mmol/l)	2.8 (1.4)	0.91 (0.81–0.96)
Ferritin (ng/ml)	1246 (1600)	0.96 (0.92–0.98)
NSE (µg/l)	35.8 (45.5)	0.93 (0.87–0.96)
Protein S100 (µg/l)	1.7 (1.42)	0.95 (0.90–0.98)
IL-6 (pg/ml)	3053 (4346)	0.94 (0.88–0.97)

CI, confidence interval; IL-6, interleukin 6; NSE, neuron-specific enolase; SD, standard deviation

In contrast, comparative measurements for cellularity had a very low correlation between the proximal and distal port sampling. The red and white blood cell counts were significantly lower in supernatant samples when compared with the proximal counterpart. In addition, the quality of supernatant cytology smears was insufficient for morphological workup. Given the good agreement of NSE measurements, profound in vitro hemolysis and white cell lysis are unlikely. We therefore assume that CSF-repellant properties of the CSF filter system that are supposed to prevent ascending infections do cause an inconsistent downstream drainage of cells into the overflow system. In addition, intraventricular gravity sedimentation of cells could also affect passive drainage of cells into the overflow chamber. Further statistical or graphical comparisons were therefore avoided. Due to the installed filter, our results are not directly comparable to the results by Wong [4], who found a high correlation for white blood cell count, if the entire CSF-volume of the drip chamber is collected.

The decisive aspect to be answered remains whether, and if so, in which indication, analysis of the supernatant from the distal overflow system may replace direct sampling from the proximal port in clinical practice. For reliable assessment of neurodestruction, analysis for both NSE and protein S100 was feasible in our study. Ferritin, a sensitive diagnostic parameter for detection of suspected SAH, was also reliably quantified from CSF supernatant. According to the Centers for Disease Control and Prevention criteria of ventriculitis, total cell count, glucose, and total protein concentration define the laboratory findings of ventriculitis. In addition, high lactate concentrations are commonly used as a surrogate parameter for developing ventriculitis. Based on our study results, it can be stated that glucose, lactate, and total protein can be reliably determined. In addition to these typical “ventriculitis markers,” IL-6 in CSF could be determined from the supernatant and was previously included in a prediction model for drain-associated ventriculitis and might play an increasing role in the future [5]. However, in case of abnormalities, a proximal sample for cell count and cytology analysis becomes indispensable.

This study demonstrates that CSF supernatant from the overflow system can be used for diagnostic workup with various biochemical parameters that are requested in several clinical conditions. Our findings indicate that other noncellularity parameters may also be assayed from CSF supernatant, for example, small molecules for therapeutic drug monitoring purposes. Harnessing CSF from the collection chamber may also come in handy for clinical studies, without the need of sampling from the proximal port. It should be noted, however, that this study was conducted with a rather small patient cohort. Larger studies are therefore required to elucidate and confirm our preliminary findings. As with any diagnostic procedure, CSF supernatant analysis from the overflow system must be validated separately for different EVD systems and preanalytical variables at each medical center.

## Data Availability

All data generated during this study are included in this article.
